# Green Valorization of Two-Phase Olive Pomace via Pressurized Liquid Extraction: Process Optimization, Comprehensive Metabolite Analysis and Functional Applications

**DOI:** 10.3390/molecules31101569

**Published:** 2026-05-08

**Authors:** Cecilia Dauber, Victoria Olt, Alberto Valdés, Silvana Alborés, Adriana Gámbaro, Elena Ibáñez, Ignacio Vieitez

**Affiliations:** 1 Departamento de Ciencia y Tecnología de Alimentos, Facultad de Química, Universidad de la República, Av. General Flores 2124, Montevideo 11800, Uruguay; volt@fq.edu.uy (V.O.); agambaro@fq.edu.uy (A.G.); 2 Laboratory of Foodomics, Institute of Food Science Research (CIAL, Consejo Superior de Investigaciones Científicas (CSIC)), Nicolás Cabrera 9, 28049 Madrid, Spain; a.valdes@csic.es (A.V.); elena.ibanez@csic.es (E.I.); 3 Departamento de Biociencias, Facultad de Química, Universidad de la República, Av. General Flores 2124, Montevideo 11800, Uruguay; salbores@fq.edu.uy

**Keywords:** olive pomace, pressurized liquid extraction, antimicrobial activity, food preservatives, natural antioxidants

## Abstract

Olive pomace (OP) has been widely reported as a rich source of phenolic compounds with potential application as food additives with health-promoting properties. The aim of this work was to evaluate pressurized liquid extraction (PLE) as a strategy to obtain antioxidant and antimicrobial extracts from OP. Extractions were carried out in laboratory-scale equipment following a combined static/dynamic procedure. The extraction temperature (100, 120 and 140 °C) and the composition of solvent (50, 75 and 100% ethanol in water) were studied as independent variables of the process using a Face Centered Central Composite Design (α = 1). According to the fitted quadratic model (*p* < 0.05), the maximum Total Phenol Content (TPC) and Trolox Equivalent Antioxidant Capacity (TEAC) values were obtained at 120 °C using ethanol concentrations between 60 and 80%. Chemical characterization by RP/HPLC-Q-TOF MS/MS allowed the tentative identification of 37 compounds, with quinic acid being the most abundant compound under all extraction conditions, followed by elenolic acid, dimethyl-hydroxy-verbascoside, maslinic acid, hydroxy-verbascoside and oleuropein aglycone. Other secoiridoids, secoridoid derivatives, flavonoids, simple phenols and triterpenic acids were also identified. The extract obtained at 120 °C with 75% ethanol was able to protect purified sunflower oil in an accelerated oxidative stability test (Rancimat), increasing its induction period by 2.4-fold when added at 1000 mg/kg. This extract also exhibited antimicrobial activity against *S. aureus*, *B. cereus*, *S. enterica* and *S. sonnei* with a Minimum Inhibitory Concentration (MIC) of 3.6 mg/mL. These results highlight the potential of PLE olive pomace extracts as natural preservatives for food applications.

## 1. Introduction

Olive pomace (OP) is one of the main by-products resulting from the olive oil production process. It is the remaining pulpy material after removing most of the oil from the olive paste and it consists of pieces of skin, pulp, stone, and olive kernel [1]. Depending on the extraction system applied, the moisture content of this residue may vary. The two-phase separation system results in a semisolid pomace (wet olive pomace or also called “alperujo”) with a higher moisture content, up to 70%, which makes it difficult to handle or store [2]. It is estimated that for every ton of olive oil produced, 4 tons of OP are generated [3], which can result in a potential environmental problem, due to its high concentration of organic matter, oil, phenols and slightly acidic pH [4]. Considering that olive oil production has tripled over the last 60 years, reaching 2,760,000 tons in the crop year 2022/23 [5], the large amounts of OP generated during the harvest and production season around the world is a matter of great concern.

Many authors have reported the valuable source of bioactive components that OP has to offer: from phenols, carbohydrates and proteins to tocopherols, pectic polymers, triterpenic acids, peptides and squalene [6,7,8]. Among them, phenolic compounds stand out as the most studied because of their multiple health benefits which include anti-inflammatory, antibacterial and antioxidant properties [9,10,11]. The interest in their recovery lines up with the need of the food industry to search for new, innovative and low-cost sources of natural ingredients to include in the development of healthier food products with functional properties. The large amount of waste generated also demands efficient strategies to manage their disposal or re-utilization in order to develop sustainable food systems [12]. Recent literature highlights that the recovery of bioactive compounds from agri-food by-products is a key strategy to reduce environmental impact, improve resource efficiency, and promote the transition from linear to circular production systems [13]. Several extraction technologies have been previously applied to olive pomace, each with specific advantages and limitations. Ultrasound-assisted extraction (UAE) is widely used due to its ability to enhance mass transfer and reduce extraction time, although it may present limitations in scalability [14]. Supercritical fluid extraction (SFE), particularly with CO_2_, offers high selectivity and solvent-free extracts, but requires high investment [15]. On the other hand, conventional solvent extraction remains a simple and accessible technique, though it generally involves higher solvent consumption and longer extraction times [2]. In this context, pressurized liquid extraction (PLE) emerges as a promising alternative, combining reduced extraction time, lower solvent use, and high efficiency under controlled conditions, with high impact in food applications [16].

PLE employs solvent extraction at high temperatures and pressures, always above the boiling point but below their respective critical points, so that the solvent is maintained in the liquid state during the whole extraction procedure. In these particular conditions, the mass transfer rates are enhanced, while at the same time, the solvent surface tension and viscosity decrease and the solubility of analytes is increases, leading to significantly higher extraction yields when compared with conventional extractions [17]. Although this technology has been developed and used on several matrices since its first appearance in the early 1990s, very few works report the use of PLE on olive pomace. For instance, Katsinas et al. [18] proposed a defatting pretreatment of OP using supercritical CO_2_ followed by a PLE optimization using hydroalcoholic mixtures. They obtained highly concentrated phenolic extracts from OP in a 3-times shorter extraction time and with 1.6 times less solvent consumption compared to conventional methods, finding that Oleacein (3,4-DHPEA-DEDA) was the most abundant polyphenol identified. Also, Cea Pavez (2019) [19] reported a great compositional variability of PLE extracts obtained under different experimental conditions, showing the great selectivity of the technology as affected by the different parameters of extraction such as the temperature, solvent flow, ratio solvent/sample and the composition of the solvent. In this case, Optimal PLE conditions allowed an enriched hydroxytyrosol extract, which was not detected in the conventional one, to be obtained. It is important to consider that the experimental conditions should be carefully optimized for each matrix. Temperature is one of the factors with the highest influence on PLE performance, since it directly affects the physicochemical properties of the solvent [20]. Increasing the temperature may generally lead to higher extraction yields but it could also cause the degradation of thermolabile compounds or unwanted chemical reactions [17]. Regarding the solvent used for the extractions, its selection will depend mostly on the target molecules and objectives of the extraction. Solvent mixtures can increase extraction efficiency because analytes with different affinities may be extracted by interacting with two solvents simultaneously [21]. In this sense, water/ethanol mixtures are usually employed in PLE as they are considered GRAS substances (Generally Recognized as Safe) and are able to extract both lipophilic and hydrophilic phenols [22].

Therefore, this study focuses on employing PLE to recover polyphenols from non-defatted olive pomace produced via the two-phase extraction system, with the aim of evaluating their potential as functional food additives, mainly antioxidant and antimicrobial. The use of non-defatted pomace was intentionally chosen to preserve the native composition of the matrix and to evaluate a more sustainable and simplified extraction approach by avoiding additional pretreatment steps. A combination of static and dynamic mode was employed to perform the extractions, studying the effect of temperature and composition of solvent (ethanol/water mixtures) on the extraction yield and the antioxidant activity of the extracts. The extracts were characterized by high-performance liquid chromatography coupled to quadrupole-time of flight mass spectrophotometry RP/HPLC-Q-TOF MS/MS in order to identify the phenolic profile. An accelerated oxidative stability test (Rancimat) and Minimum Inhibitory Concentration (MIC) assay against different bacteria were performed for the selected extract, assessing its applicability as an antimicrobial and antioxidant agent in food products. This approach contributes to the addressing of existing gaps in the literature by providing a more integrative evaluation of extraction performance and functional properties in a minimally processed matrix.

## 2. Results and Discussion

### 2.1. Optimization of Extraction Conditions

In this study, an experimental design involving two factors with three levels each was proposed to optimize the combination of PLE process variables (temperature and solvent composition) on the extraction of antioxidant compounds from olive pomace. A total of eleven experiments were carried out, including three repetitions of the central point, to study the linear, interaction and quadratic effect of the extraction parameters on three response variables: extraction yield, Total Phenol Content and in-vitro antioxidant activity against ABTS cation radical of the extracts. The combination of experimental conditions (coded and uncoded) with their respective responses are presented in Table 1.

#### 2.1.1. Extraction Yield (%)

The extraction yield of the different experiments ranged from 18.85 to 28.25% (*w*/*w*). ANOVA results showed that a second order polynomial model could adequately represent the experimental data (*p*-value < 0.05) with an R^2^ = 0.9563. According to the prediction Equation (1), the linear term of temperature (*X*_2_) and the quadratic term of ethanol concentration (*X*_1_^2^) significantly affected the response (*p*-value < 0.05), while the interaction term (*X*_1_ × *X*_2_) was not significant. The effect of temperature is clearly observed, since the highest yields were obtained for the extractions performed at 140 °C (Table 1), suggesting that increasing the temperature promotes the desorption of analytes from the matrix to the solvent, since the intermolecular interactions that bind them are reduced [17]. Similar results were reported by other authors using PLE to extract polyphenols from apple pomace, olive pomace and avocado peels [19,23,24].

*Yield* (% *w*/*w*) = 21.126 + 0.493 *X*_1_
*+* 2.765 *X*_2_ − 0.790 *X*_1_
*X*_2_ + 4.857 *X*_1_^2^ − 0.368 *X*_2_^2^ (1)

**Figure 1:**
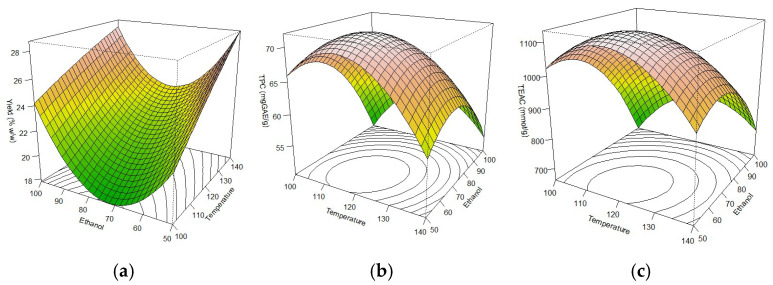
The response surface obtained for (**a**) the extraction yield, (**b**) the Total Phenol Content and (**c**) the antioxidant activity of the extracts as a function of the independent variables’ temperature and ethanol concentration in the extraction solvent.

Regarding the effect of ethanol concentration, Figure 1a shows that the extraction yield increases for extreme values (50% and 100% ethanol), showing a minimum in the intermediate region of the plot (75% ethanol). This behavior is constant for all of the range of temperatures evaluated. Although this was not initially expected, it could be explained by several factors. The fact that OP was not defatted before the extractions leads to a significant amount of remaining oil in the matrix, which contributes to the global extraction yield and is extracted mostly with higher concentrations of ethanol due to its less polar nature compared to water or ethanol/water mixtures. Nevertheless, it should be emphasized that no further studies, such as lipid quantification, were performed over the extracts to confirm this interpretation. On the other hand, the co-existence of very polar and nonpolar polyphenols may lead to higher extraction yields at the lowest or highest concentrations of ethanol in the solvent but at intermediate levels, it may occur that none of the groups of compounds dissolve optimally. Other components of the matrix such as pectic polysaccharides may also be subjected to extraction under the experimental conditions tested, mostly when higher concentrations of water are used [6]. The extraction yield, as defined in Equation (4), represents the sum of the total mass extracted from the matrix, without differentiating which families of compounds are present. Considering that each group of compounds extracted responds in a different way to changes in experimental conditions, specifically to solvent composition, it seems reasonable to think that there is no obvious response in this sense. These interpretations were formulated considering the known composition of non-defatted olive pomace and the influence of solvent polarity on the solubility of different compound families. It should also be considered that it is not necessarily the highest extraction yields that will correspond to better antioxidant properties, as discussed in the following sections.

#### 2.1.2. Total Phenol Content (TPC)

The Total Phenol Content of the extracts determined by the Folin–Ciocalteau method ranged from 41.21 to 72.36 mg GAE/g. These values are higher than reported for other green extraction procedures, like mechanical pressing [25], supercritical fluid extraction (SFE) [15] or ultrasound assisted extraction (UAE) [14], which reinforces the use of PLE as a promising alternative for the recovery of polyphenols from OP. The adjusted model for this response was significant (*p*-value < 0.05) and explained 86.6% of the experimental variability. Regarding the prediction Equation (2), the factors that significantly affected the response were the linear term ethanol concentration (*X*_1_) and the quadratic term of temperature (*X*_2_^2^), the latter being the one with the greatest impact, derived from the highest value of its coefficient. According to Figure 1b, the best results are achieved with temperatures close to 120 °C and a concentration of ethanol between 60 and 70%.

*TPC* (mg GAE/g) = 68.694 − 6.018 *X*_1_ − 1.215 *X*_2_ + 2.560 *X*_1_
*X*_2_ − 5.239 *X*_1_^2^ − 13.629 *X*_2_^2^ (2)

Several phenomena may be useful to understand the effect of temperature. Firstly, as mentioned earlier, increasing the temperature promotes the release of solutes (including phenolic compounds) from the matrix. This effect is enhanced by working under pressure conditions, which favors the matrix rupture. In addition to this, it is important to consider that a large proportion of the phenolic acids present in OP are found linked to cell-wall cellulose and lignin [26]. As temperature rises, these bonds are broken and lignin itself may be degraded giving rise to more phenolic acids [27]. The combination of these two effects leads to an increase in TPC that is observed when raising the temperature from 100 to 120 °C, reaching a maximum near the center of the temperature range studied. Beyond that point, it is likely that thermal degradation of phenolic compounds has a predominant effect, leading to a pronounced decrease in TPC for higher values of temperature (Figure 1b). Previous studies have reported that elevated temperatures can promote not only the release of bound phenolics but also their degradation through oxidation, hydrolysis, or structural transformation, particularly above optimal extraction temperatures [20,27]. In relation to the effect of ethanol concentration on TPC, the results obtained are similar to other works reported previously, where intermediate values (50–70%) provide an optimal recovery of total phenols [21,28,29,30]. According to Nguyen-Kim (2021) [30], at an ethanol concentration close to 70%, the presence of water in solvent causes the matrix to be moderately swelled, thus facilitating the permeation of solvent into the material. Higher concentrations of ethanol in the extraction solvent lead to a decrease in the TPC of the extracts. As a general rule, polar phenols are more easily extracted in hydro-ethanolic mixtures (25–75 mL ethanol/100 mL) instead of absolute ethanol [22] because a wider range of polarity is covered. Nevertheless, it must be considered that optimal ethanol concentration used in PLE is specific for each family of polyphenols, such as phenolic acids, flavanols, flavonols and stilbenes [31].

#### 2.1.3. Antioxidant Activity by ABTS Cation Radical Assay

The antioxidant activity of the extracts showed a behavior similar to that observed for TPC. The lowest value (623.2 µmol TE/g) was observed for the extract obtained with 100% ethanol and 100 °C, while the highest values (average 1116 µmol TE/g) correspond to the extracts obtained with 75% ethanol and 120 °C, matching the conditions of the central point of the experimental design proposed. Experimental data was satisfactorily adjusted to a second-degree polynomial model (R^2^ = 0.8896). According to the ANOVA, the terms with statistical significance were the linear and quadratic term of ethanol concentration and the quadratic term of temperature (Equation (3)). These results indicate that antioxidant activity is closely related to the total phenolic content (Pearson Index = 0.89), since both response variables are affected in the same way by the independent factors studied, which can also be noted when comparing their response surfaces (Figure 1b,c).

*TEAC* (µmol TE/g) = 1094.03 − 134.23 *X*_1_ − 0.983 *X*_2_
*+* 47.25 *X*_1_
*X*_2_ − 121.12 *X*_1_^2^ − 132.46 *X*_2_^2^ (3)

#### 2.1.4. Selection of Optimized Extraction Conditions

Based on the results obtained from the response surface analysis, the extraction conditions of 120 °C and 75% ethanol in water were selected for subsequent experiments. This combination represents the central point of the experimental design, for which experimental replications were performed, providing high reliability and reproducibility of the results. At these conditions, the extracts exhibited the highest total phenolic content (68–72 mg GAE/g) and antioxidant activity (≈1100 µmol TE/g), while maintaining a satisfactory extraction yield (≈20%). The temperature of 120 °C ensures an efficient release of phenolic compounds from the OP matrix without promoting thermal degradation, which was observed at 140 °C. Similarly, an ethanol concentration of 75% provides an optimal balance between solvent polarity and extraction efficiency. Therefore, these conditions were considered the most suitable compromise between extraction efficiency, antioxidant potential, and experimental robustness.

### 2.2. Chemical Characterization of Olive Pomace Extracts

A total of 37 compounds were tentatively identified in the OP extracts, all of them previously reported in olive products and related by-products. Table 2 summarizes the list of tentative compounds, which are ordered based on their retention time, and includes the corresponding MS/MS fragment ions obtained in negative ESI mode together with specific literature references supporting its identification. This analysis was intended for qualitative and semi-quantitative profiling, and reported differences should be interpreted as indicative trends rather than absolute quantitative variations.

As a first observation, it is worth noting that all the compounds described were present in all the extracts, with only minor differences observed in their distribution among them. Tentatively identified compounds were grouped into organic acids (peaks 1 and 2), simple phenols (peaks 3 and 4), phenylpropanoids/phenolic acids (peaks 6 and 9), phenylpropanoid glycosides (peaks 10, 11, 12, 15, 20, 21 and 25), secoiridoids/iridoids (peaks 5, 7, 8, 14, 16, 19, 23, 27, 28, 29, 31, 33 and 35), flavonoids (peaks 13, 17, 18, 22, 24, 26, 30 and 34), lignans (peak 32) and triterpenic acids (peaks 36 and 37), with secoiridoids and phenylpropanoid glycosides being the most representative families. Regardless of the extraction temperature or solvent composition, quinic acid (peak 1) was the major compound in all the extracts, accounting for 27.6–33.4% of the total identified area. Its MS/MS fragmentation pattern included the deprotonated molecular ion [M-H]^−^ at *m*/*z* 191 and other characteristic fragment ions (at *m*/*z* 85, 59 and 93). The relative abundance of this organic acid across all the conditions is consistent with its highly polar nature, which gives the molecule a strong affinity for hydroalcoholic mixtures. Also, quinic acid may originate from the hydrolysis of more complex phenolic esters naturally present in olive pomace (e.g., chlorogenic acid), a process that could be favored by the high temperatures used during the extractions, thus increasing its relative contribution to the overall composition of the extracts. These results are consistent with those reported by Cea-Pávez (2019) [19] who also found quinic acid as one of the major phenolic compounds in OP extracts obtained by PLE.

Elenolic acid (peak 14), the second most abundant compound tentatively identified, accounted from 11.7 to 20.9% to the total relative area. This non-phenolic compound constitutes the iridoid part of some of the most characteristic olive secondary metabolites, namely secoiridoids [32]. Its relatively high abundance suggests that the applied extraction conditions may have promoted the partial degradation or hydrolysis of more complex secoiridoids originally present in the matrix, like oleuropein. This is consistent with the fact that oleuropein, although it was detected in the extracts (peak 27), contributed less than 1% to their overall composition. The same behavior was observed for secologanoside (peak 7). On the other hand, oleuropein aglycone, a known product of hydrolysis of oleuropein derived from the loss of the glucose moiety, was detected in the extracts (peak 35) with a relative contribution of 3.6–6.1%. The simultaneous presence of both intact secoiridoids (at low concentrations) together with elenolic acid and oleuropein aglycone suggests, therefore, that PLE conditions promoted partial hydrolysis and degradation of complex secoiridoids into lower molecular weight derivatives. This behavior can be mainly attributed to the elevated temperatures applied during PLE, which enhance reaction kinetics and may facilitate the cleavage of ester and glycosidic bonds. In addition, the presence of water in the ethanol/water mixtures likely contributes to hydrolytic processes by increasing water activity and promoting bond cleavage.

Other tentatively identified compounds also belonging to the secoiridoid family were two isomers of nüzhenide (peaks 16 and 19) and nüzhenide 11-methyl oleoside (peak 31) which have been described as the main phenolic compounds in olive seed [33]. Although they have also been found in OP extracts [25], their contribution was relatively low. Among the phenylpropanoid glycosides, hydroxy-verbascoside isomer 1 (peak 10) and dimethyl-hydroxy-verbascoside isomer 1 (peak 20) were the most abundant. The difference of 28 Da observed between their deprotonated molecular ions supports the presence of two additional methyl groups in the demethylated derivative. Also, some characteristic fragment ions were found for both compounds (at *m*/*z* 179, 161, 459) reflecting their common structural backbone.

Flavonoids, a group of compounds recognized for its important contribution to the radical scavenging activity of olive derivatives [34] were represented by glycosides of apigenin, quercetin and luteolin as well as their aglycone forms, except for quercetin, which was only present as quercetin-rutinoside and quercetin-3-rhamnoside. Among this group, luteolin (peak 30) stood out as the most relevant in terms of its relative contribution, up to 4.5%, followed by luteolin glucoside (peak 17), up to 1.4%. The rest of the compounds were present only marginally (<0.5%).

Compound 36 was tentatively identified as maslinic acid, a triterpenoid with demonstrated biological activities such as antioxidant, anti-inflammatory, anti-viral and anti-cancer [35]. Its relative contribution to the composition of OP extracts was quite stable for all experimental conditions, being one of the ten most abundant compounds present. On the other hand, oleanolic acid (peak 37), the other triterpenic acid identified in the OP extracts, only reached a maximum of 1.6%.

Regarding the specific chemical profile of the selected extract (120 °C and 75% ethanol), quinic acid was the predominant compound (29.2%), followed by elenolic acid (15.9%) and dimethyl-hydroxyverbascoside isomer 1 (8.2%). Other relevant constituents included maslinic acid (5.6%), hydroxyverbascoside isomer 1 (5.1%), oleuropein aglycone (5.0%), luteolin (4.0%), nüzhenide 11-methyl oleoside (3.6%), decarboxymethyl elenolic acid ester (3.0%) and hydroxytyrosol (2.6%). Together, these ten compounds accounted for approximately 82% of the total identified peak area. This complex phytochemical composition reflects the potential of OP extracts as a rich source of bioactive compounds that may act synergistically and contribute to their antimicrobial and antioxidant properties. This hypothesis is based on literature evidence rather than a demonstrated correlation within this study [36,37].

**Table 2:** Tentative identification of compounds in all PLE extracts determined by RP/HPLC-Q-TOF MS/MS).

Peak	RT (min)	Tentative Identification *	Molecular Formula	[M-H]^−^ Theoretical	[M-H]^−^ Experimental	Error (ppm)	MS^2^ Product Ions	Relative Contribution (% Area)	References
1	0.666	**Quinic acid**	C_7_H_12_O_6_	191.0556	191.0572	−8.56	85.03 (100), 191.06 (81), 59.01 (37), 93.04 (23), 87.01 (21), 127.04 (13)	27.6–33.4	[25,38,39,40,41]
2	0.733	Malic acid	C_4_H_6_O_5_	133.0137	133.0147	−7.52	72.99 (100), 59.02 (59), 71.01 (45)	1.3–2.2	[39]
3	2.232	**Hydroxytyrosol**	C_8_H_10_O_3_	153.05517	153.0562	−6.73	123.05 (100), 122.04 (55), 109.03 (13), 95.05 (12)	1.6–3.7	[25,38,39,40,41,42]
4	2.302	Hydroxytyrosol glucoside	C_14_H_20_O_8_	315.107995	315.1082	−0.65	153.06 (100), 123.05 (52)	0.6–0.7	[25,38,39,40,42]
5	3.250	**Decarboxymethyl elenolic acid ester**	C_10_H_14_O_4_	197.081385	197.0823	−4.64	123.05 (100), 151.04 (39), 122.04 (37), 95.05 (9)	2.2–4.1	[39]
6	3.321	Coumaric acid glucoside	C_15_H_18_O_8_	325.092345	325.0930	−2.01	163.04 (100), 119.05 (78)	0.2–0.3	[40,42]
7	3.353	Secologanoside	C_16_H_22_O_11_	389.10839	389.1092	−2.08	69.03 (100), 71.01 (47), 165.06 (43), 121.07 (42), 271.12 (30), 209.04 (29), 59.01 (28)	0.5–1.2	[25,38,39]
8	3.405	Methyl glucooleoside	C_23_H_34_O_16_	601.153543	601.1536	−0.09	403.12 (100), 601.17 (28), 89.03 (24), 131.04 (22), 404.13 (21), 181.07 (21)	0.2–0.5	NIST Database
9	3.522	Caffeic acid	C_9_H_8_O_4_	179.034435	179.0355	−5.95	135.04 (100), 136.05 (23), 134.03 (8)	0.1–0.2	[25,40,42]
10	4.559	**Hydroxy-** **verbascoside 1**	C_29_H_36_O_16_	639.192515	639.1928	−0.45	639.19 (100), 621.18 (35), 622.18 (18), 179.03 (18), 161.02 (11), 459.15 (6)	4.1–6.1	[25,39,40]
11	4.947	Hydroxy- verbascoside 2	C_29_H_36_O_16_	639.192515	639.1921	0.65	639.19 (100), 621.18 (30), 475.15 (8), 161.02 (5)	0.2–1.0	[25,39,40]
12	5.101	Hydroxy- verbascoside 3	C_29_H_36_O_16_	639.192515	639.1926	−0.13	639.19 (100), 621.18 (22), 142.00 (9), 161.02 (9)	0.1–0.4	[25,39,40]
13	5.272	Quercetin rutinoside	C_27_H_30_O_16_	609.145565	609.1465	−1.53	609.15 (100), 300.03 (13), 301.04 (11)	0.3–0.9	[25,38,39,40,41]
14	5.350	**Elenolic acid**	C_11_H_14_O_6_	241.071215	241.0731	−7.82	139.00 (100), 95.05 (89), 69.00 (88), 111.01 (56), 101.03 (52), 67.02 (31)	11.7–20.9	[25,39,40,42]
15	5.380	Verbascoside	C_29_H_36_O_15_	623.1976	623.1984	−1.28	623.20 (100), 461.17 (8), 161.02 (8), 315.11 (7)	1.0–1.7	[25,38,39,40,41,42]
16	5.454	Nüzhenide 1	C_31_H_42_O_17_	685.23438	685.2352	−1.20	523.18 (100), 443.16 (75), 685.24 (54), 524.19 (50), 453.14 (43), 421.15 (32), 444.16 (31), 447.10 (22)	0.3–0.4	[25,33,40,42]
17	5.464	Luteolin glucoside	C_21_H_20_O_11_	447.09274	447.0938	−2.37	447.09 (100), 285.04 (69), 284.03 (16), 286.04 (7)	1.0–1.4	[25,38,39,40,41]
18	5.558	Luteolin rutinoside	C_27_H_30_O_15_	593.15065	593.1512	−0.93	593.15 (100), 285.04 (10), 545.18 (6), 96.96 (6)	0.3–0.5	[25,38,39,40,41,42]
19	5.701	Nüzhenide 2	C_31_H_42_O_17_	685.23438	685.2357	−1.93	453.14 (100), 523.18 (91), 421.15 (40), 685.23 (40), 623.20 (33), 454.14 (29), 223.06 (17)	1.1–1.4	[25,33,40,42]
20	5.720	**Dimethyl-hydroxy-** **verbascoside 1**	C_31_H_40_O_16_	667.2238	667.2255	−2.53	667.23 (100), 621.18 (76), 622.19 (28), 179.03 (18), 161.03 (15), 459.15 (10), 487.15 (10)	6.3–10.1	[39]
21	5.741	Isoverbascoside	C_29_H_36_O_15_	623.1976	623.1978	−0.32	621.20 (100), 161.02 (19), 179.03 (17), 459.15 (6), 151.04 (5)	0.4–0.9	[25,38,39]
22	5.926	Quercetin 3- rhamnoside	C_21_H_20_O_11_	447.0927	447.0930	−0.58	285.04 (100), 301.04 (27), 286.04 (22), 447.10 (19), 300.03 (18), 167.03 (13)	0.1–0.3	NIST Database
23	5.936	Caffeoyl-6′- secologanoside	C_25_H_28_O_14_	551.1401	551.1406	−0.93	551.14 (100), 161.02 (63), 507.15 (42), 389.11 (24), 167.03 (22), 67.02 (21)	0.3–0.9	[25,38,39,40,42]
24	6.034	Apigenin glucoside	C_21_H_20_O_10_	431.0978	431.0987	−2.03	431.10 (100), 268.04 (74), 269.05 (72)	0.1	[39,41]
25	6.047	**Dimethyl-hydroxy-** **verbascoside 2**	C_31_H_40_O_16_	667.2238	667.2246	−1.18	621.18 (100), 667.22 (91), 622.19 (22), 487.15 (14), 623.18 (10), 251.05 (8), 179.04 (7)	0.8–2.6	[39]
26	6.054	Apigenin rutinoside	C_27_H_30_O_14_	577.1557	577.1591	−5.83	577.16 (100), 269.04 (51), 415.14 (48), 431.10 (33)	0.1	[25,39,41]
27	6.421	Oleuropein	C_25_H_32_O_13_	539.1765	539.1773	−1.54	377.12 (100), 275.09 (74), 539.17 (63), 403.12 (58), 276.06 (35)	0.3–0.6	[25,38,39,40,41,42]
28	6.500	**Coumaroyl-** **secologanoside**	C_25_H_28_O_13_	535.1452	535.1455	−0.62	145.03 (100), 535.15 (87), 491.15 (53), 345.12 (22), 163.04 (20), 121.07 (19)	2.5–4.8	[25,38,39,40,42]
29	6.973	Lucidumoside C	C_27_H_36_O_14_	583.2027	583.2029	−0.37	151.04 (100),61.99 (69), 537.16 (48), 403.12 (46), 179.05 (36), 583.24 (33)	0.5–0.9	[25,38,41]
30	7.228	**Luteolin**	C_15_H_10_O_6_	285.0399	285.0402	−1.00	285.04 (100), 151.00 (14), 133.03 (13), 217.05 (6)	3.0–4.5	[25,38,39,40,41]
31	7.363	Nüzhenide 11- methyl oleoside	C_48_H_64_O_27_	1071.3557	1071.3554	0.26	685.23 (100), 1071.35 (91), 686.24 (53), 771.23 (24), 523.18 (17), 453.14 (7)	0.4–1.6	[33]
32	7.553	Lignan derivative	C_20_H_18_O_5_	337.1076	337.1082	−1.78	307.06 (100), 322.08 (44), 290.06 (15), 245.88 (14)	0.2–0.4	[38]
33	7.652	6′-O-[2.6-Dimethyl-8-hydroxy-2-octenoyloxy]-secologanoside	C_26_H_38_O_13_	557.2234	557.224	−1.04	557.23 (100), 121.06 (26), 227.13 (19), 513.23 (18), 165.05 (11), 183.07 (11), 185.12 (10)	0.8–1.2	[25,38,39,41]
34	8.028	Apigenin	C_15_H_10_O_5_	269.045	269.0456	−2.23	269.04 (100), 117.03 (55)	0.2–0.3	[40]
35	8.224	**Oleuropein aglycone**	C_19_H_22_O_8_	377.1236	377.1249	−3.33	139.04 (100), 95.05 (93), 111.01 (62), 149.02 (58), 139.00 (54), 275.06 (49), 127.04 (38)	3.5–6.1	[25,38,39,40,42]
36	9.737	**Maslinic acid**	C_30_H_48_O_4_	471.3474	471.3494	−4.17	471.35 (100)	4.5–6.4	[25,38]
37	10.401	Oleanolic acid	C_30_H_48_O_3_	455.35252	455.3536	−2.37	455.35 (100)	1.0–1.6	[38]

* The names highlighted in “bold” represent the ten compounds with the highest relative contribution to the composition of the extracts, accounting for approximately 80–85% of the total identified area in all the cases.

### 2.3. Oxidative Stability Evaluation

Accelerated oxidative stability tests provide insight into the potential of newly developed antioxidants to enhance the oxidative stability of lipid matrices. This can give an approximation on how efficiently such additives can perform when incorporated into vegetable oils or other food systems to prevent oxidation. With this purpose, the selected OP extract was incorporated into purified sunflower oil at two different concentrations: 500 and 1000 mg/kg. A control sample (pure SFO) was also subjected to the experiment, as well as a sample with 100 mg/kg BHT, a synthetic antioxidant commonly used in the food industry. The induction periods of the four samples studied are reported in Table 3. The addition of OP extract led to significantly higher (*p* < 0.05) IPs in both cases, with a protection factor (PF) of 1.6 and 2.4 for 500 and 1000 mg/kg, respectively, showing a dose-dependent performance. Also, the extract at 1000 ppm extended the induction period beyond that achieved by 100 ppm BHT, validating the replacement of synthetic antioxidants with naturally obtained OP extract. Günal & Turan (2018) [43] observed an increment of 1.4-fold in the IP of sunflower oil when adding 1000 mg/kg of an olive pomace extract that contained a similar concentration of total phenols to the one used for this study. These results reinforce the potential of PLE as an effective and innovative technology for obtaining antioxidant-rich extracts from olive pomace with enhanced protective performance in lipid systems.

### 2.4. Antimicrobial Activity

Olive products and by-products have been widely reported to exert antimicrobial activity against pathogenic bacteria and toxigenic fungi [13,44,45,46]. However, information regarding the antimicrobial properties of olive pomace extracts specifically obtained by PLE remains limited. In this sense, the ability of the selected extract (120 °C, 75% EtOH) to inhibit the growth of three Gram-positive bacteria and four Gram-negative bacteria was tested by Minimum Inhibitory Concentration (MIC) assay.

The extract inhibited the growth of *S. aureus*, *S. enterica*, *B. cereus* and *S. sonnei*, at 3.6 mg/mL (Table 4), while *B. subtilis*, *E. coli* and *K. pneumoniae*, were not inhibited at the concentrations evaluated. This differential susceptibility can be related to both the bacterial cell envelope structure and the extract chemical composition. From a structural perspective, Gram-positive bacteria are generally more susceptible to phenolic compounds due to the absence of an outer membrane, facilitating the interaction of bioactive molecules with the cytoplasmic membrane. The thick peptidoglycan layer does not represent a strong permeability barrier, allowing bioactive molecules to interact more easily with the cytoplasmic membrane and intracellular targets [47]. This could explain the inhibition observed for *S. aureus* and *B. cereus*. In contrast, Gram-negative bacteria possess an outer membrane enriched in lipopolysaccharides (LPS), which acts as a selective permeability barrier and limits the diffusion of several phenolic and secoiridoid-derived compounds [48]. This structural feature likely accounts for the lack of activity against *E. coli* and *K. pneumoniae*. Nevertheless, the inhibition of *S. enterica* and *S. sonnei*, both Gram-negative, indicates that susceptibility cannot be explained solely by Gram classification. Differences in the outer membrane composition, the porin expression, the efflux systems, and the membrane permeability among the strains may influence the access and intracellular accumulation of phenolic compounds.

When comparing our results with the literature, a high variability in MIC values is evident. Nunes (2021) [49] reported MIC values for *E. coli* ranging from 62.5 to 125 mg/mL, while lower MIC values were observed for *S. aureus* (31.25–125 mg/mL), using extracts obtained by applying physical pressure on homogenized OP with subsequent separation of liquid and solid fractions. Similarly, Fotiadou (2024) [50] using 50% acetone or 50% ethanol extracts, described MIC values between 2.5 to 10.0 mg/mL for *E. coli* and *B. subtillis*. On the other hand, Zhao (2023) [51] reported much lower MIC values (0.25–2.1 mg/mL) for *E. coli* when evaluating different fractions of an aqueous OP extract. These discrepancies confirm that antimicrobial activity strongly depends on the extraction methodology, the solvent polarity, the fractionation strategy, and consequently, the chemical profile.

Importantly, these differences should also be interpreted considering that MIC values for plant extracts and complex natural matrices are typically several orders of magnitude higher than those of conventional antibiotics. While antibiotics usually exhibit MICs in the range of 0.01–10 µg/mL, plant extracts generally show activity in the µg/mL to mg/mL range [52]. In our study, gentamicin was used as a reference antibiotic and yielded MIC values in the µg/mL range, supporting this comparison. Consistently, numerous studies report MIC values in the mg/mL range for natural extracts, including hydromethanolic plant extracts with MICs between 0.1 and 12.8 mg/mL [53] and even higher values (7.8–62.5 mg/mL) against resistant clinical isolates [54], indicating that MICs of a few mg/mL are not uncommon for crude extracts.

Finally, the practical relevance of the observed MIC values should be interpreted in light of both the complexity of the extract and its intended application. Olive pomace extracts are phenolic-rich mixtures (e.g., hydroxytyrosol, tyrosol, and secoiridoids) that typically exhibit moderate antimicrobial activity, often within the mg/mL range, as also reported for olive by-products and related matrices [49]. These extracts are not intended for systemic antimicrobial therapy but rather for applications such as functional food ingredients or natural preservatives. In this context, higher effective concentrations are technologically feasible, and their value lies in their multifunctionality (e.g., combined antioxidant and antimicrobial activities) rather than in low MIC values alone. This perspective is consistent with previous reports highlighting that the efficacy of phenolic-rich plant extracts in food systems strongly depends on the matrix and application conditions [55].

Considering the chemical composition of the selected OP-PLE extract, its antimicrobial activity is unlikely to be attributable to a single compound. The presence of low molecular weight phenolics such as hydroxytyrosol, elenolic acid derivatives, and oleuropein aglycone (Table 2) suggests a multi-target mode of action. Serra (2008) [56] demonstrated that natural OP extracts exhibited higher antimicrobial activity than isolated hydroxytyrosol, quercetin or oleuropein tested individually, supporting the hypothesis of synergistic interactions among constituents. In this context, the inhibition of *S. enterica* and *S. sonnei*, despite their Gram-negative structure, could be associated with synergistic effects among phenolic compounds that enhance the membrane permeability or potentiate intracellular damage. Such combinatorial interactions may overcome, at least partially, the permeability barrier imposed by the outer membrane.

Overall, our findings support the concept that antimicrobial activity of OP-PLE extracts results from a complex interplay between bacterial structural features and extract chemical composition, with synergistic interactions among phenolics likely playing a key role. The observed variability across studies further highlights the importance of detailed chemical characterization when comparing the antimicrobial efficacy of olive pomace extracts obtained under different extraction conditions. Taken together, these results support the potential application of the OP extracts obtained by PLE as natural food preservatives. Their ability to inhibit relevant foodborne pathogens reinforces their value as functional ingredients derived from agro-industrial by-products. Nevertheless, further research is required to evaluate their effectiveness in real food matrices.

## 3. Materials and Methods

### 3.1. Samples

Wet olive pomace from Arbequina cultivar was collected directly from an olive mill (two-phase system) located in Maldonado Department (Uruguay) and transported in plastic bags under refrigerated conditions to the laboratory where it was dried at 40 °C for 22 h. Dried OP (moisture < 1% *w*/*w*) was ground in a domestic mill and stored at −18 °C until analysis. A unified sample was prepared from three different batches of dried OP collected on different days during the same harvesting period. This approach aimed to minimize the variability associated with raw material heterogeneity, and the replicates performed correspond to technical replicates, as all experiments were conducted using this homogenized sample. The drying conditions were selected as a mild treatment to minimize thermal degradation of phenolic compounds while ensuring adequate moisture removal for sample stabilization. Previous studies have shown that although freeze-drying provides the highest retention of phenolic compounds, conventional drying methods at controlled temperatures can achieve comparable results when appropriately applied, with the process time being a critical factor [57]. In addition, drying at moderate temperatures has been widely used for olive pomace processing and allows preservation of antioxidant properties while maintaining the process simplicity, since freeze-drying is considerably more expensive and less scalable for industrial applications due to its high energy consumption and operational costs. [2].

### 3.2. Pressurized Liquid Extractions

PLE extractions were carried out in laboratory-scale equipment (Singularity Extraction Technologies, Campinas, Brazil). Briefly, the solvent was pumped from a solvent reservoir to the extraction cell using an HPLC pump (Shimadzu LC-6A, Kyoto, Japan) coupled to the system. For each experiment, 13 g of dried OP were loaded into the extraction cell (25 mL capacity) together with approximately 10 mL of solvent. A heat-up step was performed before the extraction until the target temperature was reached. The extractor is equipped with three independently controlled heating systems: (1) pre-heating of the solvent, (2) cell heating and (3) micrometric valve heating. Systems 1 and 2 were set at the target extraction temperature while system 3 was maintained at 80 °C to prevent clogging. Once the conditions of pressure and temperature were achieved for each run, a static extraction step of 10 min was performed, followed by a dynamic extraction step of 30 min with a constant flow of 2 mL/min (total solvent volume of 60 mL). This time was defined considering a kinetic study with the matrix in which aliquots of the extract were collected at subsequent times and analyzed to determine the accumulated phenols extracted (Figure S1). The solvent loaded with the extract was collected after depressurization through a micrometric valve. After the extraction time was completed, the extract was subjected to solvent evaporation under vacuum in a rotary evaporator (Buchi Rotavapor R-114, BÜCHI Labortechnik AG, Flawil, Switzerland) and stored at −18 °C for further analysis.

### 3.3. Experimental Design

The effect of two independent factors, namely the temperature (*X*_1_) and the ethanol concentration (*v*/*v*) in an ethanol/water mixture (*X*_2_) was studied using a Face Centered Central Composite Design (α = 1). These two factors have been previously identified as the most relevant in terms of the extraction yield and the phenol recovery in PLE extractions [58]. On the other hand, the extraction pressure was set at a constant value of 10 MPa, since previous studies reported the null influence of the extraction pressure beyond the point at which the solvent is maintained liquid [59]. The temperature ranged from 100 °C to 140 °C, while the ethanol concentration ranged from 50% to 100%. A total of 11 runs were carried out, including three repetitions of the central point (120 °C, 75% ethanol) to get an estimate of the experimental error. Three response variables were considered: the global extraction yield (%), the Total Phenol Content of the extract (TPC) and the Trolox equivalent antioxidant activity of the extract (TEAC). The experimental data for each response was adjusted to a second order polynomial regression model. An analysis of variance (ANOVA) was performed to determine if the model was statistically significant (*p* < 0.05) and to obtain the prediction equation for each variable identifying the terms that significantly affected the response. A graphical representation of the response surface was obtained with “rsm package”, from the statistical software “R version 4.2.1” [60].

### 3.4. Extract Analysis

#### 3.4.1. Global Extraction Yield

The extraction yield (%m/m) was calculated for each experiment as expressed in Equation (4), where the dried extract mass corresponds to the total mass of extract after the solvent evaporation.

Global yield (%) = (dried extract mass (g))/(dried sample mass (g)) × 100(4)

#### 3.4.2. Total Phenolics and Antioxidant Activity

For the TPC and TEAC determination, the solid extracts were prepared by extracting with a methanol/water solution (80:20). This solvent system has been extensively applied in the literature for the analysis of phenolics in plant and agro-industrial matrices [61]. Briefly, 1000 µL of extraction solution were added to 80 mg of dried extract and shaken for 1 min in a Hinotec QL-866 vortex shaker (Ningbo, China). Each tube was placed in an ultrasonic bath for 10 min (temperature during the sonication was controlled and maintained at approximately 25 °C to avoid potential degradation of phenolic compounds), then centrifuged (6000 rpm, 10 min) and the supernatant was filtered with a 0.45 µm PVDF syringe filter. The TPC and TEAC were determined by Folin–Ciocalteau and ABTS+. cation radical assay respectively, as described by Dauber (2022) [15]. The results for the TPC were calculated using a calibration curve with gallic acid and expressed as mg GAE/g extract while for TEAC, Trolox was used as standard and results were expressed as µmol Trolox equivalents/g extract. Standards for the calibration curves were provided by Sigma-Aldrich, St. Louis, MO, USA.

#### 3.4.3. Reverse Phase Liquid Chromatography-Quadrupole-Time of Flight Mass Spectrophotometry (RP/HPLC-Q-TOF MS/MS) Analysis

Dried PLE extracts were dissolved in pure ethanol to a final concentration of 5 mg/mL. Then, the samples were vortexed for 30 s, centrifuged at 14,800 rpm for 5 min at 4 °C and the supernatants were collected and stored at −80 °C until analysis. Aliquots of 2 μL were injected (in triplicates) into an HPLC (model 1290) coupled to a quadrupole Q-TOF (6540 series) equipped with a Jet Stream thermal orthogonal ESI source, all from Agilent Technologies (Waldbronn, Germany). The compounds were separated using an Agilent ZORBAX Eclipse Plus C18 analytical column (100 mm length × 2.1 mm id, 1.8 μm particle size) and an Agilent C18 guard column (0.5 cm × 2.1 mm, 1.8 μm particle size). Milli-Q water with 0.1% formic acid was used as mobile phase A while acetonitrile (ACN) with 0.1% formic acid was used as mobile phase (B). The column temperature was held at 40 °C, the flow rate was set to 0.5 mL/min and the following chromatographic gradient was used: 0–30% B in 7 min; 30–80% B in 2 min; 80–100% B in 2 min; 100% B for 2 min; 100–0% B in 1 min; and 0% B for 3 min. The mass spectrometer was operated in negative mode using the following parameters: *m*/*z* range: 40 to 1700; capillary voltage: 3000 V; fragmentor voltage 110 V; skimmer voltage: 45; octapole voltage 750 V; nebulizer pressure: 40 psig; drying gas flow rate: 8 L/min; drying gas temperature: 300 °C; sheath gas flow: 11 L/min; sheath gas temperature: 350 °C. MS/MS analyses were performed using collision energies of 20 and 40 V. For proper mass accuracy, spectra were corrected using ions at *m*/*z* 119.0363 (C_5_H_4_N_4_) and 966.0007 (C_18_H_18_O_6_N_3_P_3_F_24_ + formate), simultaneously pumped into the ionization source. For data processing, LC-MS raw data files were converted to ABF format using Reifycs Abf Converter (v1.3.8802) and chromatograms were analyzed using MS-DIAL v4.9 software to obtain the relative abundances (as area under the curve, AUC). Finally, the tentative identification of the compounds was performed by comparison with MS/MS spectra from NIST, LipidBLAST and MoNA databases, or with data reported in the literature.

#### 3.4.4. Accelerated Oxidative Stability Test

In order to assess the potential of the selected OP extract to protect a food product from oxidative damage, a commercial sunflower oil (SFO) with no synthetic antioxidants declared was purchased from the local market and purified by passing it through a column packed with aluminum oxide to remove residual tocopherols that remained after the refining process [62]. The OP extract was added to the purified oil in two concentrations: 500 and 1000 mg/kg. For comparison purposes, a sample with 100 mg/kg of butylhydroxytoluene (BHT), a synthetic antioxidant commonly used in the food industry, was also evaluated. The three samples together with a control sample (pure SFO) were subjected to an oxidative stability test according to the AOCS cd 12b-92 official technique [63] at 100 °C using a 873 Biodiesel Rancimat equipment (Metrohm, Herisau, Switzerland). The induction period (IP) was determined for each sample, and a relative protection factor was calculated as the ratio between the IP of the sample with the extract or the BHT and the IP of the control sample. The experiment was performed in triplicate for each sample.

#### 3.4.5. Antimicrobial Activity

The selected extract was evaluated as a growth inhibitor of some common bacteria of concern in the food industry. The Minimum Inhibitory Concentration (MIC) was determined by the microdilution technique [64] against the following bacteria: *Escherichia coli* ATCC 25922, *Staphylococcus aureus* ATCC 6538, *Bacillus subtilis* ATCC 6633, *Bacillus cereus* CCMG14, *Salmonella enterica* ATCC 14028, *Shigella soneii* CCMG12015 and *Klebsiella pneumoniae* CCMG12716. A suspension (~1.5 × 10^8^ cells/mL; 0.5 McFarland) was prepared from a fresh (24 h) culture grown on Nutrient Agar. This suspension was seeded into sterile 96-well microplates (300 μL capacity, MicroWell, NUNC, Thermo Fisher Scientific, Waltham, MA, USA) containing Mueller–Hinton broth, along with serial dilutions of the extract prepared in 20% (*v*/*v*) dimethyl sulfoxide (DMSO) and gentamicin as the reference antimicrobial. Positive controls (growth control) were prepared containing inoculum and sterile DMSO 20% solution assuring no antimicrobial effect. Negative controls were also prepared with sterile broth to ensure they were not contaminated. After incubation at 37 °C during 24 h, the plates were revealed using triphenyl tetrazolium chloride (TTC). The MIC was determined as the lowest extract concentration (antimicrobial agent) that inhibited the visible growth of the microorganism after 24 h.

### 3.5. Statistical Analysis

Analyses of TPC and TEAC were performed in triplicate for each extract, each undergoing the full extraction and analytical procedure. All the data obtained from the experimental design for each response variable was fitted to quadratic models using the statistical software R [60] and the adequacy of the models was assessed by analysis of variance (ANOVA). Model terms were considered significant at a 95% confidence level (*p* < 0.05). Response surface plots were generated using the “rsm” package in R. Experimental data were analyzed by analysis of variance (ANOVA) to determine the statistical significance of the factors. Significant differences between samples were determined by the Tukey test, considering a significance level of *p* < 0.05.

## 4. Conclusions

This study demonstrates the potential of PLE as an efficient and selective green technology for the valorization of OP, a highly abundant by-product of the olive oil industry. By optimizing the temperature and solvent composition, high-value extracts rich in phenolic compounds with high antioxidant and antimicrobial properties were obtained without the need for prior defatting or toxic solvents, reinforcing the novelty of the proposed approach. From a scientific and technological perspective, this work provides new evidence on how moderate PLE conditions (120 °C, 75% ethanol) allow a balanced recovery of diverse bioactive families, including phenylpropanoid glycosides, secoiridoids, flavonoids, and triterpenic acids. The compositional profile suggests that PLE promotes partial transformation of complex olive secoiridoids into lower molecular weight derivatives, which may contribute to the enhanced functional performance of the extracts.

Regarding industrial applicability, the selected extract significantly improved the oxidative stability of sunflower oil and exhibited antimicrobial activity against relevant foodborne pathogens, supporting its potential use as a natural preservative in food formulations. A key limitation of this study is the reliance on semi-quantitative profiling and the use of a single homogenized sample, which restricts the ability to establish direct relationships between composition and bioactivity. Therefore, future work should include absolute quantification of target compounds, evaluation of different raw material batches, and validation of the extracts in real food systems to better assess their applicability. Future studies should also directly compare defatted and non-defatted matrices to clarify their impact on extraction efficiency and bioactivity.

## Figures and Tables

**Table 1:** Experimental design of extractions and results obtained for extraction yield, TPC and antioxidant activity of OP extracts. (X_1_: ethanol concentration, X_2_: temperature).

Run	Coded Factors		Uncoded Variables		Response Variables		
	X_1_	X_2_	% EtOH	T (°C)	Yield (%)	TPC (mgGAE/g)	TEAC (µmol/g)
1	1	−1	100	100	24.02	41.21	623.2
2	1	0	100	120	26.42	62.37	881.6
3	1	1	100	140	28.25	41.47	746.3
4	0	−1	75	100	18.85	52.81	959.5
5	0	0	75	120	19.97	67.57	1134.5
6	0	1	75	140	23.82	55.24	896.4
7	−1	−1	50	100	20.82	64.34	1062.7
8	−1	0	50	120	26.70	62.46	997.0
9	−1	1	50	140	28.21	54.36	996.8
10	0	0	75	120	20.71	68.23	1120.0
11	0	0	75	120	20.45	72.36	1094.8

**Table 3:** Induction period (minutes) of SFO with addition of BHT or PLE olive pomace extract at different concentrations.

Sample	IP (min)	PF *
SFO	72 ± 4 ^d^	1.0
SFO + 100 ppm BHT	149 ± 6 ^b^	2.1
SFO + 500 ppm PLE extract	116 ± 5 ^c^	1.6
SFO + 1000 ppm PLE extract	172 ± 5 ^a^	2.4

* PF: Protection Factor. ppm: parts per million (mg/kg). The mean values with different letters in each column are significantly different (*p* < 0.05), as determined by the Tukey test.

**Table 4:** Minimum Inhibitory Concentration (mg/mL) of selected OP-PLE extract against different bacteria.

Microorganism	MIC (mg/mL)
Gram-positive	
*Staphylococcus aureus*	3.6
*Bacillus cereus*	3.6
*Bacillus subtilis*	>3.6
Gram-negative	
*Salmonella enterica*	3.6
*Shigella sonnei*	3.6
*Klebsiella pneumoniae*	>3.6
*Escherichia coli*	>3.6

## Data Availability

Dataset available on request from the authors.

## Supplementary Materials

The following supporting information can be downloaded at: https://www.mdpi.com/article/10.3390/molecules31101569/s1, Figure S1. [A] Cumulative yield (%m/m) and [B] cumulative phenol yield (mg GAE/g OP) VS solvent-to-sample ratio (S/F) in the dynamic extraction step. Extraction time was set at S/F=4, equivalent to 30 minutes of solvent flow at 2 mL/min.

## Abbreviations

The following abbreviations are used in this manuscript:

ABTS	2,2′-azino-bis-(3-ethylbenzothiazoline-6-sulfonic) acid
ANOVA	Analysis of Variance
ASE	Accelerated Solvent Extraction
AUC	Area Under Curve
BHT	Butylated hydroxytoluene
CCD	Central Composite Design
ESI	Electrospray Ionization
GAE	Gallic Acid Equivalent
GRAS	Generally Recognized as Safe
HPLC	High-Performance Liquid Chromatography
IP	Induction Period
LPS	Lipopolysaccharides
MIC	Minimum Inhibitory Concentration
MoNA	MassBank of North America
MS	Mass Spectrometry
NIST	National Institute of Standards and Technology
OP	Olive Pomace
PF	Protection Factor
PLE	Pressurized Liquid Extraction
Q-TOF	Quadrupole–Time of Flight
SFE	Supercritical Fluid Extraction
SFO	Sunflower Oil
TE	Trolox Equivalent
TEAC	Trolox Equivalent Antioxidant Capacity
TPC	Total Phenol Content
UAE	Ultrasound Assisted Extraction
