# Supplementary material for: Green Valorization of Two-Phase Olive Pomace via Pressurized Liquid Extraction: Process Optimization, Comprehensive Metabolite Analysis and Functional Applications
Source: Molecules. 2026 May 8;31(10):1569. doi: 10.3390/molecules31101569 (PMC13209781; doi:10.3390/molecules31101569)
Supplement: Supplementary file 1 [file molecules-31-01569-s001.zip › molecules-4281806-supplementary.pdf]

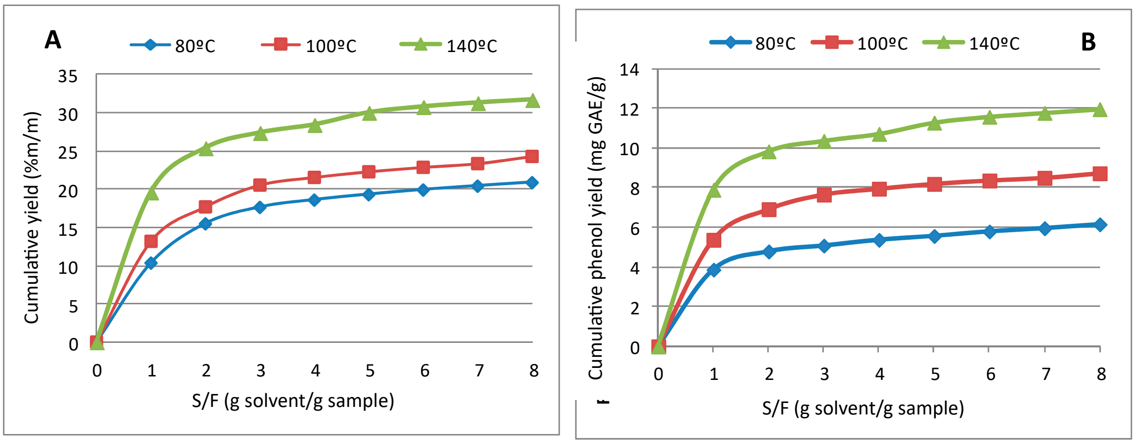

Figure S1: [A] Cumulative yield (%m/m) and [B] cumulative phenol yield (mg GAE/g OP) VS solvent-to-sample ratio (S/F) in the dynamic extraction step. Extraction time was set at S/F=4, equivalent to 30 minutes of solvent flow at 2 mL/min.
